# Developmental Coordination Disorder (DCD): Relevance for Clinical Psychologists in Europe

**DOI:** 10.32872/cpe.4165

**Published:** 2022-06-30

**Authors:** Emily J. Meachon, Martina Zemp, Georg W. Alpers

**Affiliations:** 1 Department of Psychology, School of Social Sciences, University of Mannheim, Mannheim, Germany; 2 Department of Clinical and Health Psychology, University of Vienna, Vienna, Austria; Philipps-University of Marburg, Marburg, Germany

**Keywords:** dyspraxia, neurodevelopmental disorders, motor coordination, clinical practice, psychotherapy

## Abstract

**Background:**

Developmental Coordination Disorder (DCD) is a common neurodevelopmental disorder primarily characterized by fine and gross motor coordination difficulties. Yet, many aspects remain unclear regarding the clinical presentation of secondary symptoms and their implications for Clinical Psychology. Therefore, the purpose of this review is to provide an update about the current understanding of DCD for clinical psychologists and psychotherapists across Europe, particularly based on new insights stemming from the last decade of research.

**Method:**

We provide a narrative review of articles published in the last decade on the topic of DCD, and relevant aspects to clinical psychologist, including lesser known aspects of DCD (e.g., executive functions, psychological consequences, and adult DCD).

**Results:**

DCD is a highly prevalent, disruptive, and complex disorder, which should be investigated further in many areas (e.g., co-occurrence to ADHD). Existing evidence points toward a key role of executive functioning difficulties at all ages. Most patients report secondary psychological problems, but little headway has been made in examining the effectiveness of psychotherapy for DCD.

**Conclusions:**

Insights and remaining research gaps are discussed. It is critical for psychologists and clinical researchers to raise awareness for DCD, take note of the growing literature, and foster continued interdisciplinary approaches to research and treatment of DCD.

## Highlights

- Awareness about Developmental Coordination Disorder (DCD) is low among some European Psychologists.
- Growing knowledge about DCD should be disseminated among psychotherapists.

*Tanja*^1^1This case is based on collective experiences of individuals with DCD, and is not based on any one real person.
*is a 20-year-old female from Germany who studies part time at university and has a part-time job as a store manager. She has noticed she takes much longer than her peers to type her papers, and she often struggles to pay attention to long lectures. When she was younger, she had trouble learning how to ride a bike, and struggled to grip her pencils correctly, however, she improved both skills during childhood. She has found that her struggles to pay attention and difficulties with typing are becoming problems at work, but her classmates and colleagues do not seem to notice she is struggling. As the demands of her job and studies increased, her difficulties have become extremely burdensome. Therefore, Tanja is seeking psychotherapy to manage her stress.*

At first glance, some clinicians may suspect the patient has Attention-Deficit/Hyperactivity Disorder (ADHD) based on the characteristic problems with sustained attention. However, she also exemplifies several hallmark symptoms of Developmental Coordination Disorder (DCD). A correct diagnosis in Tanja’s case could be critical because treatment for ADHD may require different strategies (i.e., medication). Considering the common misconceptions and lack of knowledge surrounding DCD, it is important clinicians treating complex cases like these are aware of the current clinical picture of DCD.

## Key Aspects of Developmental Coordination Disorder

DCD is a neurodevelopmental disorder with primary deficits in fine and gross motor coordination (American Psychiatric Association, 2013). The DSM-5 criteria for a DCD diagnosis include: (1) the acquisition and execution of motor skills and related coordination are below what is expected based on age, (2) the deficits of motor skill and coordination significantly interfere with daily life in the domains of self-care, scholastics, work, leisure, and play, (3) the symptoms began in childhood, and (4) the deficits cannot be better explained by any other condition (e.g., cerebral palsy or neurodegenerative disorder; American Psychiatric Association, 2013; see Table 1). DCD has a profound impact on the lives of individuals suffering from the disorder.

**Table 1:** Diagnostic Criteria and Examples of Symptoms of DCD

Diagnostic Manual / Criteria	Practical Example	Recommendations
DSM 5: Developmental Coordination Disorder		
(A) the acquisition and execution of motor skills and related coordination are below what is expected based on age	The individual might have taken longer to learn to crawl, walk, ride a bike, write, kick a football, climb or descend stairs, etc. They might have also learned motor skills but struggle to execute them in a coordinated fashion.	In children, the MABC-2 (Henderson et al., 2007) can be used to objectively assess motor functions in comparison to same-aged peers (in a percentile score based on age-band). In adults, MABC-2 can be used loosely, a self-report by the patient of novel motor experiences in adulthood might be considered, e.g., a new skill in the workplace or school: typing, driving.
(B) the deficits of motor skill and coordination significantly interfere with daily life in the domains of self-care, scholastics, work, leisure, and play	The individual might avoid socialization, or team sports, in fear of embarrassment for lack of coordination.	Screen for impact of motor skills on daily life, and other psychosocial factor (e.g., co-occurring anxiety, depression).
(C) the symptoms began in childhood	–	If patient is an adult at the time of assessment, the Adult DCD Checklist (ADC; Kirby et al., 2010) section 1 can be used as a proxy for symptoms in childhood.
(D) the deficits cannot be better explained by any other condition	Patient should not have Cerebral Palsy, Huntington’s Disease, acquired brain injury, difficulties related to surgery, etc.	Complete diagnostic history, including physical, mental, and genetic conditions, should be considered.
ICD-10: Specific Developmental Disorder of Motor Function (F82)		
(1) A disorder with primary deficits of motor coordination	As listed in DSM 5 criterion (A) above.	As listed in DSM 5 criterion (A) above.
(2) Impairments in fine and gross motor coordination	General difficulties might involve fine motor tasks such as trouble gripping objects, poor handwriting, challenges typing on a keyboard. Difficulties might also involve gross motor functions, such as, trouble walking in a coordinated manner, frequently tripping over or bumping into objects, difficulties kicking or catching a ball.	As listed in DSM 5 criterion (A) above.
(3) Not better explained by an intellectual disability or acquired neurological disorder	Patient should not have Disorder of Intellectual Development, Cerebral Palsy, Huntington’s Disease, acquired brain injury, difficulties related to surgery, etc.	As listed in DSM criterion (D) above. Potential rationale for IQ testing.

*Note.* The ICD-11 “Developmental Motor Coordination Disorder” lists symptoms entirely in line with the DSM 5, adding that symptoms must begin in childhood. Notably, the different name contradicts nomenclature standards set out by DCD experts (see Blank et al., 2019) and the patient preferred name “Dyspraxia.” Diagnostic criteria are summarized from the latest guidelines of each diagnostic manual (DSM-5-TR; American Psychiatric Association, 2022; ICD-11; World Health Organization, 2020). In the new DSM-5-TR, DCD is listed under a further subcategory entitled “motor disorders.

Accumulating research highlights the psychological effects of DCD symptoms still remain unclear (e.g., Kirby et al., 2013; Tal Saban & Kirby, 2018; Zwicker et al., 2018) and executive functioning differences may be present (e.g., Bernardi et al., 2018; Sartori et al., 2020). Furthermore, there is a lack of established gold standard diagnostic procedure for adults with DCD despite increasing evidence that motor symptoms and psychosocial consequences continue into adulthood in most cases (Purcell et al., 2015; Tal Saban & Kirby, 2018).

**Figure 1:**
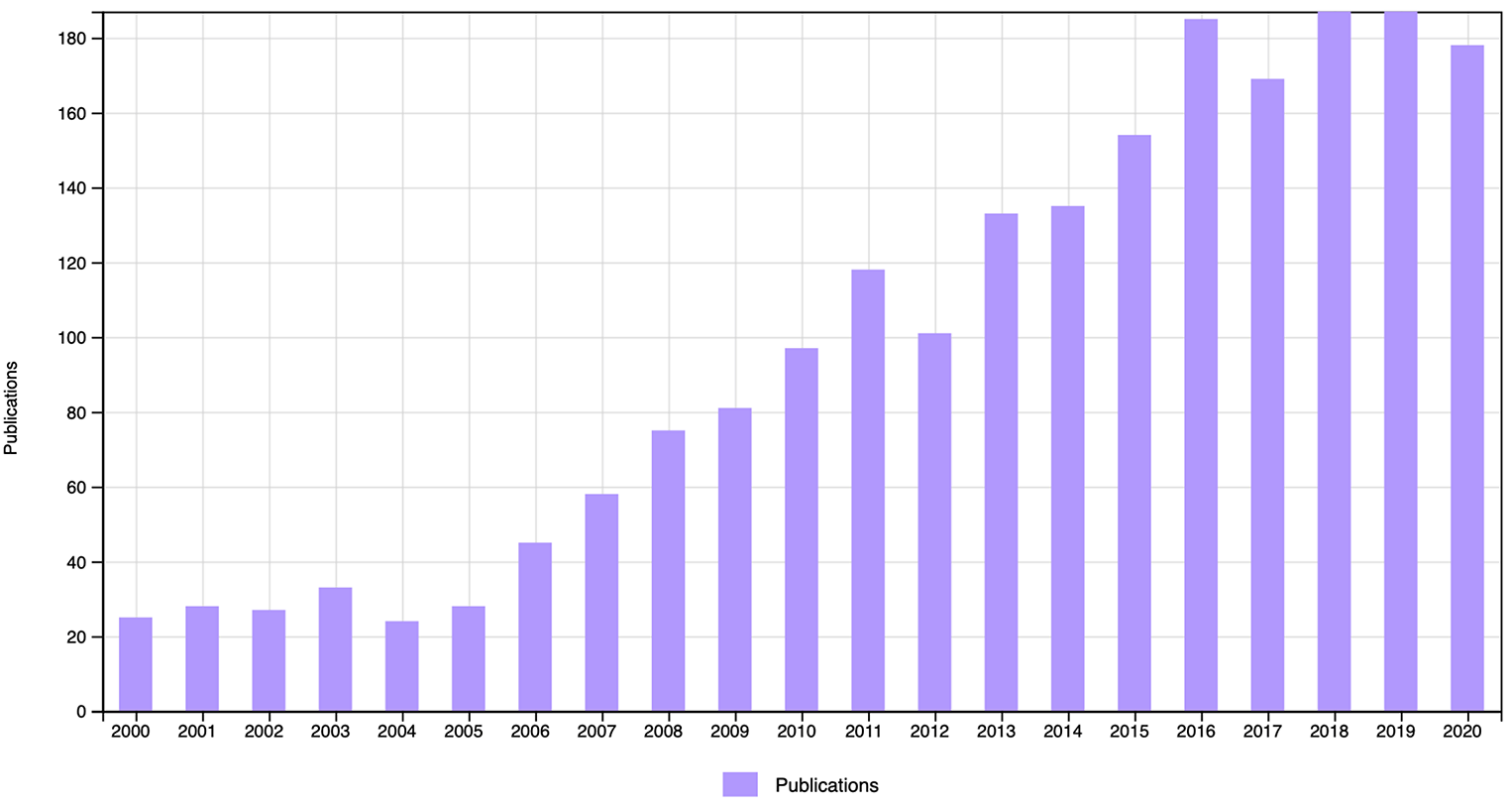
Publications With the Term “Developmental Coordination Disorder” in the Title, Abstract, or Key Words From 2000-2020 *Note.* A) a total of *k* = 2,068 articles were retrieved from the search in Web of Science in June 2021. While many search topics have increased in research volume over the years, as a closer comparison, the search term “Attention Deficit Hyperactivity Disorder” returned *k* = 28,533 articles from the same time period with at least *k* = 1,000 per year from 2009 on, and *k* = 2,480 in 2020 alone; exceeding the number in one year for all DCD articles across 20 years.

DCD is a common neurodevelopmental disorder, with a prevalence frequently cited as 5% (Blank et al., 2019). Despite this, DCD has received minimal attention in research, especially compared to other neurodevelopmental disorders (see Figure 1; Bishop, 2010). Even child and adolescent psychiatrists have been reported to profess poor general knowledge of DCD (Wilson et al., 2013). This alludes to a history of potentially overlooking individuals with DCD.

While the number of publications and citations for papers about DCD is still far behind comparable conditions (e.g., ADHD), there has been a promising increase in publications over the last decade (see Figure 1). In addition, international guidelines for most aspects of DCD were recently released for health care professionals of all fields (Blank et al., 2019). The guidelines solidify that DCD is a unique condition to be recognized by psychologists and offer important insights. Therefore, in this narrative review, we (1) extend upon these guidelines to include an overview on the current state of lesser understood features of DCD (e.g., executive functions, co-occuring ADHD, adult DCD), and (2) highlight available resources specifically for European psychologists (e.g., tools available in various European languages). We include recent insights with research primarily published in the last decade to provide an up-to-date overview of DCD.

## Method

The present review is narrative in nature and included evidence from several systematic searches on the PsycINFO and Web of Science databases in November 2020. Search terms included “Developmental Coordination Disorder,” “Dyspraxia,” and “DCD” in all sections, and some subsections required separate extensive searches. For example, screening tools for DCD were searched by name (i.e., MABC-2; BOT-2; DCD-Q; Adult Developmental Coordination Disorders/Dyspraxia Checklist; AAC-Q). In order to find a comprehensive list of these tools in all European languages, additional searches were conducted on Google Scholar with the name of the language as an additional search term for each of the screening tools (see Table 2). Eligible records were those published between 2009-2020, which were reviews, expert consensus papers, empirical papers, and meta-analyses regarding DCD and relevant aspects to clinical psychology (e.g., psychosocial consequences; executive functions; DCD in adults).

**Table 2:** Published and Validated Screening Tools for Developmental Coordination Disorder in European Languages

Language	Motor Screening Tests		Questionnaires for Children		Questionnaires for Adults	
	MABC-2 *(ages 3 to 16)*	BOT-2 *(ages 4-21)*	DCD-Q *(ages 5-15)*	Little DCD-Q *(ages 3-4)*	ADC *(ages 17 - 42)*	AAC-Q *(ages 16-35)*
Language Relevant to Europe						
Czech	Psotta et al., 2012	N/A	N/A	N/A	N/A	N/A
Danish	Reported available by Blank et al., 2019	N/A	Milidou et al., 2015	N/A	N/A	N/A
Dutch	Schoemaker et al., 2012	N/A	DCDQ-NL; Schoemaker et al., 2006	LDCDQ-NL; Cantell et al., 2019	N/A	N/A
English	UK, Henderson et al., 2007^a^	USA, Bruininks & Bruininks, 2005^a^	Wilson et al., 2009 ^a^	Canadian, Wilson et al., 2015	UK, Kirby et al., 2010^a^	Tal Saban et al., 2012 ^a^
Flemish	N/A	N/A	N/A	Reported available by Rihtman et al., 2015 L-DCD-Q-VL Moret et al., 2019	N/A	N/A
French	Marquet-Doléac et al., 2016	N/A	DCDQ-FE: Ray-Kaeser et al., 2019	Reported available by Rihtman et al., 2015	N/A	N/A
German	Petermann, 2008	Blank et al., 2014	DCDQ-G; Kennedy-Behr et al., 2013	Reported available by Rihtman et al., 2015	Meachon et al., 2022	N/A
Greek	Ellinoudis et al., 2011	N/A	N/A^b^	N/A	N/A	N/A
Italian	Zoia et al., 2019	N/A	Caravale et al., 2015	N/A	N/A	N/A
Maltese	N/A	N/A	Camilleri et al., 2020	N/A	N/A	N/A
Norwegian	Holm et al., 2013	N/A	N/A	N/A	N/A	N/A
Polish	N/A	N/A	DCDQ’07-PL; Nowak, 2016	N/A	N/A	N/A
Slovenian	Reported available by Blank et al., 2019	N/A	Tercon et al., 2015	Reported available by Rihtman et al., 2015	N/A	N/A
Spanish	age band 1: Niño-Cruz et al., 2019	for 4-7 years old children: Serrano-Gómez & Correa-Bautista, 2015	Salamanca et al., 2012	Reported available by Rihtman et al., 2015	N/A	Delgado-Lobete et al., 2021
Swedish	Reported available by Blank et al., 2019	N/A	Iwar, 2015	N/A	N/A	N/A
Other Relevant Languages						
Hebrew	N/A	N/A	Version 1: Bar-Ilan Traub et al., 2005	LDCD-Q; Rihtman et al., 2011^a^	Kirby et al., 2010 ^a^	N/A
Portuguese (Brazil)	Valentini et al., 2014; Capistrano et al., 2015	Okuda et al., 2019	Prado et al., 2009	Reported available by Rihtman et al., 2015	N/A	N/A
Turkish	N/A	N/A	Yildirim et al., 2019	N/A	N/A	N/A

*Note.* MABC-2: Movement Assessment Battery for Children 2nd edition; BOT-2: Bruininks-Osteretsky Test of Motor Proficiency; DCD-Q: Developmental Coordination Disorder Questionnaire; ADC: Adult Developmental Coordination Disorders/Dyspraxia Checklist; AAC-Q: Adolescents and Adults Coordination Questionnaire; N/A indicates tool is not yet available in the listed language.

^a^Denotes original version. ^b^Listed in DCDQ administration manual, but not elsewhere.

### Clinical Presentation and Secondary Psychosocial Consequences

DCD has a lifetime prognosis with major symptoms including difficulties with planning and execution of fine motor (e.g., sketching) and gross motor coordination (e.g., riding a bicycle). As described in the DSM 5, individuals with DCD can appear to be generally clumsy, and often have delays in reaching motor milestones compared to their peers (American Psychiatric Association, 2013). Examples of this can be very evident, such as having to spend longer than other children in learning how to hold a pencil, or subtler, such as having more trouble learning to play a musical instrument in school than other children. Notably, research in the last decade has provided increasing evidence that symptoms of DCD extend beyond motor coordination. More specifically, impaired executive functions (i.e., inhibition, cognitive control, working memory, and related processes such as attention) can be recognized as a prominent feature of DCD (Bernardi et al., 2018; Leonard & Hill, 2015; Sartori et al., 2020). However, neither the DSM-5 nor the ICD-11 consider these as potential symptoms of DCD (Purcell et al., 2015; see Table 1). Furthermore, the specific symptom profiles and the extent to which executive function impairments in DCD can be attributed to co-occurring conditions (e.g., attention and inhibition difficulties typical to ADHD) remains unclear (Blank et al., 2019).

A combination of executive functioning and motor coordination difficulties may result in a plethora of consequences and challenges for individuals with DCD in all stages of life. Recent research has suggested core symptoms of DCD likely entail secondary psychological problems, such as decreased quality of life, lower self-esteem, impaired social relationships compared to typically developing peers (e.g., Tal Saban & Kirby, 2018; Zwicker et al., 2018). Internalizing symptoms in the form of secondary anxiety and depression may often occur as a consequence of DCD (Draghi et al., 2020; Kirby et al., 2013; Mancini et al., 2019; Omer et al., 2019; Rigoli & Piek, 2016), which should be of concern in psychotherapy. More research is needed to understand the specificity of these features as they are known in similar conditions (e.g., ADHD). Consequences of DCD also include a risk for obesity, cardiovascular problems, reduced fitness ability, and worse self-reported general health compared to typically developing peers (Cairney et al., 2017; Joshi et al., 2015; Kirby et al., 2013).

### Existing Evidence of DCD Prevalence and Etiology

Despite the often stated DCD prevalence rate of 5% (Blank et al., 2012; Blank et al., 2019), prevalence in some in many countries is not clear. Among existing estimates is that 1-19% of school-aged children in the UK suffer from DCD (Zwicker et al., 2012), but more recent estimates are around 10% in samples from the US and 24% from Brazil based on a study children in these regions (Valentini et al., 2017). While the prevalence rate in adults is not known, DCD is estimated to persist into adulthood in 30-70% of cases (Tal Saban & Kirby, 2018). In addition, a recent cross-sectional analysis of children in Spain estimates the prevalence of high risk for DCD is about 12% (Delgado-Lobete et al., 2019). Differences in prevalence estimates still vary greatly between existing studies, possibly due to a variance in identification of DCD.

Previous research has estimated that DCD occurs three to seven times more often in males than females (Zwicker et al., 2012), with recent evidence of a more equal gender ratio in a Brazilian sample (Valentini et al., 2017). However, these gender differences are not necessarily universal, as some recent research has found a more equal ratio between gender in Brazil (Valentini et al., 2017). These gender differences may also be a consequence of bias in detection of symptoms or referral bias, as has occurred for similar neurodevelopmental disorders such as ADHD (Young et al., 2020). Beyond gender differences, recent research found that left-handedness is nearly twice as prevalent among those with DCD as it is for typically developing controls (Darvik et al., 2018). Further research has yet to explore the underlying mechanisms in this phenomenon.

Relatively little is known about the causes of DCD. Compelling evidence for a 70% heritability estimate for DCD was calculated with a population of Swedish twin pairs (Lichtenstein et al., 2010). Low birth weight and premature birth are also predictors of DCD, particularly among males (Spittle et al., 2021; Zwicker et al., 2012). While little is known about risk factors for DCD aside from being male and preterm (van Hoorn et al., 2021), some research on neurodevelopmental disorders in general suggests there may be additional links to family income in addition to low birthweight and premature birth (e.g., Carlsson et al., 2021).

### DCD With Co-Occurring ADHD or Autism Spectrum Disorder

Among the various challenges in the diagnosis and detection of DCD are its co-occurring conditions (Cleaton & Kirby, 2018). For instance, DCD and Attention-Deficit/Hyperactivity Disorder (ADHD) have a particularly high co-occurrence of about 50% (Blank et al., 2019). Given the symptomatic overlaps, including motor impairments in ADHD (Kaiser et al., 2015), and attention, inhibition, and hyperactivity sometimes observed in DCD (Harrowell et al., 2018; Wilson et al., 2020), some have speculated whether DCD might be a subtype of ADHD. While concrete evidence for this assumption remains limited to date, more research speaks for a unique pathology in DCD (e.g., in the genetic profile, Pearsall-Jones et al., 2009; physiological responding, Goulardins et al., 2015; neural mechanisms, Meachon et al., 2021). This has also been supported by findings for unique functional pathways in co-occurring DCD and ADHD as opposed to just one disorder (McLeod et al., 2014). It is important that this co-occurrence receives more scientific attention in the future to identify not only the extent to which the clinical symptoms but also their endophenotypes overlap (e.g., Conzelmann et al., 2009). This may help to prevent misdiagnosis, given the many similarities between DCD and ADHD. One simple step researchers and clinicians can take to work toward this goal is to screen for DCD when working with patients who have ADHD (Lange, 2018), and vice versa.

Another common co-occurrence is Autism Spectrum Disorder (ASD; Caçola et al., 2017). ASD can be diagnosed as a co-occurring disorder of DCD since the DSM-5, and researchers are just beginning to explore the co-occurring diagnosis. Unlike ADHD, existing literature clearly supports that the difficulties sourcing from DCD or ASD are unique (Paquet et al., 2019). For example, a systematic review of DCD and ASD behavioral outcomes primarily found clear differences between DCD and ASD (Caçola et al., 2017). Thus, it can be assumed that co-occurring cases of DCD and ASD present a much more complex symptom profile than DCD or ASD alone

### Available Screening Tools for DCD in European Languages

In the screening and diagnostic process for DCD, the current best practice is to ensure all four major DSM-5 criteria for diagnosis are met. There are various tests and screening tools which European clinicians can use to identify if a diagnosis of DCD should be considered. The most common tools relevant to European psychologists will be highlighted in this section.

Motor skill assessment is crucial to establish meeting the first criterion for a DCD diagnosis in the DSM-5: that motor skills are below the expected development compared to same-age peers. While there are many tools which can be used to assess motor skills (see Cancer et al., 2020 for an overview of other motor screening tools for children), two of the most common screening tools used to assess risk for DCD are the *Movement Assessment Battery for Children* (MABC-2; Henderson et al., 2007) and the *Bruininks-Oseretsky Test of motor proficiency* (BOT-2; Bruininks & Bruininks, 2005). The MABC-2 was developed in English to identify probable DCD in children aged 3 to 16, and is available in Dutch (Schoemaker et al., 2012), German (Petermann, 2008), Italian (Zoia et al., 2019), Greek (Ellinoudis et al., 2011), Norwegian (Holm et al., 2013), and Spanish (age band 1 validated by Niño-Cruz et al., 2019; see Table 2). In addition, some researchers have reported using the MABC-2 but do not reference a validated translation or test of the psychometric properties of the reported language used (e.g., Danish, Slovenian, Swedish; Blank et al., 2019).

The BOT-2 was designed in English as a motor competency test for broader populations among children from 4 to young adults of 21 years old, and available in German (Blank et al., 2014), and Spanish (validated for 4-7 years old children by Serrano-Gómez and Correa-Bautista, 2015). The BOT-2 can reportedly be used to diagnose individuals of any language group, because it uses motor-skill games independent of language (Baharudin et al., 2020), however, its norms should be extended beyond what is now exclusively based on US norms. For example, recent research on the ecological validity of the German BOT-2 showed it strongly relates to other relevant fine motor skills and some gross motor skills, however subtests for bilateral coordination and balance do not have clear ecological validity (e.g., to sports and bike riding) among German children (Vinçon et al., 2017). Notably, the concerns of translation (i.e., for the MABC-2 checklist) and norms is also prevalent with MABC-2, which was developed with UK samples. Some slight differences were observed between British norms and those of other nationalities tested on the MABC-2, suggesting the consideration norms for motor tests be adapted to specific countries, even within Europe (Barnett, 2014; Zoia et al., 2019). Given the age cutoffs, caution should be taken in the interpretation of scores for adolescents and adults, and should not outweigh assessment of the other diagnostic criteria for DCD.

There are several questionnaires which can be used to assess the second and third DSM-5 criterion regarding persistent interruptions of symptoms and presence of symptoms in childhood. For children, the *Developmental Coordination Disorder Questionnaire* is a popular parent-report measure of DCD symptoms developed in English (Wilson et al., 2009). The DCD-Q has been translated and validated into many languages spoken in Europe (see Table 2, including German, (DCDQ-G; Kennedy-Behr et al., 2013), Dutch (DCDQ-NL; Schoemaker et al., 2006), Italian (Caravale et al., 2015), Spanish (Salamanca et al., 2012), Danish (Milidou et al., 2015), and French-European (DCDQ-FE: Ray-Kaeser et al., 2019). In addition, a version to indicate DCD in young children (ages 3-4) exists, known as the *Little Developmental Coordination Disorder Questionnaire* developed in Hebrew (LDCD-Q; Rihtman et al., 2011) and translated into English (LDCDQ-CA; Wilson et al., 2015) and Dutch (LDCDQ-NL; Cantell et al., 2019). The LDCDQ was also translated into many European languages (Rihtman et al., 2015; see Table 1), however validation studies to confirm these translations have not yet been published. Notably, in adolescent populations, parents were less accurate in identifying motor competencies than their adolescent children’s self-reports (Timler et al., 2018), but to our knowledge, there is no evidence if this is the same in children versus parent-reports. Therefore, parent-reports should be used with caution in older children, and should be accompanied by in-depth assessment of the adolescents themselves.

To gain better insight into the daily life interruptions in adulthood, the self-report *Adult Developmental Co-ordination Disorders/Dyspraxia Checklist* (ADC) was developed and validated in English and Hebrew to detect probable cases of DCD in individuals 16 years and older (Kirby et al., 2010). The ADC was also recently translated into German (Meachon et al., 2022) and reevaluated for potential to screen for motor and executive functioning parameters of DCD (Meachon et al., 2022). In addition to the ADC, Tal Saban et al. (2012) developed the Adolescents and Adults Coordination Questionnaire (AAC-Q) as a shorter-form self-report tool to screen for DCD compared to the ADC. The AAC-Q was developed in English (Tal Saban et al., 2012) and recently translated into Spanish (Delgado-Lobete et al., 2021). While retrospective diagnosis of DCD in adulthood is certainly possible, it must be on the premise that symptom experiences began in childhood. There is currently no gold standard motor assessment tool for screening in adults.

In accordance with the final criterion of the DSM-5 for DCD, causes of clumsiness or differences in gait from other medical conditions or brain injury must be ruled out. Contrary to the exclusion criteria of intellectual disorders listed in the DSM-5 and ICD-10 (DSM-5; American Psychiatric Association, 2013; ICD-10; World Health Organization, 2016), children with DCD may score lower than average on some or all domains of IQ tests due to interruptions in motor processing and perception (Jaščenoka & Petermann, 2018). Recent consensus established that IQ score cutoffs should not prevent the diagnosis of DCD (Blank et al., 2019). More research is needed to conclude if this is consistent across the development and into adulthood.

### DCD in Adolescents and Adults

Most of the existing research on DCD examines populations of affected children rather than adolescents and adults, even though a majority of adults with DCD continue to experience symptom-related difficulties in their daily lives (Tal Saban & Kirby, 2018). This mirrors a pattern observed in ADHD research, which primarily focuses on child and adolescent populations (Targum & Adler, 2014). The history of overlooking adult populations could be for strictly following diagnostic criteria for DCD (i.e., it must begin in childhood; American Psychiatric Association, 2013). Other possibilities might include (1) the lack of assessment tools for adults, (2) the complex phenotype in adulthood (e.g., co-occurring conditions, symptom progression), and (3) the heterogeneous compensatory strategies adults develop to deal with their motor constraints. Concerning the latter, compensatory strategies may mask symptoms for simple motor tasks (e.g., hand rotation task; Wilmut, 2017). This should be carefully considered in the diagnostic process for DCD, especially for adults with DCD who were not diagnosed in childhood.

Adults with DCD often struggle with difficulties in psychosocial domains, executive functioning, physical fitness, time management, and organization (e.g., Kirby et al., 2013; Kirby et al., 2011; Tal Saban & Kirby, 2018). In general, underlying mechanisms of DCD are not likely change across the lifespan, however the context, experience of the individual, and compensation may change. For example, motor challenges and difficulty with distance estimation may manifest in adulthood as problems in learning to drive or even crossing the road compared to typical adults (Kirby et al., 2010; Wilmut & Purcell, 2020). While the most relevant DCD symptoms for adults may vary interindividually, symptoms that are less easily detected or treated could become more problematic in adulthood. For example, executive functioning challenges were among the most commonly reported daily concerns for adults with suspected DCD (Purcell et al., 2015), a concern that might not be addressed in traditional physical training to treat symptoms of DCD.

There are also relationships between DCD and increased cognitive difficulties, fatigue, and somatic symptoms compared to a control group, albeit findings are based on cross-sectional data (Thomas & Christopher, 2018). Because of the considerable overlaps between DCD and ADHD that can also be present in adulthood, future research should work toward identifying the specific symptom profiles of DCD and ADHD.

Despite considerable research gaps on adult populations with DCD, some recent research has investigated DCD in emerging adults between the ages of 16 to 25 (e.g., Kirby et al., 2011). This group may still be dependent on their parents but are working toward independence and identity exploration (Tal Saban & Kirby, 2018). Due to the major life changes this age group commonly faces, it may be at risk for experiencing heightened difficulty in coping with DCD symptoms, and should be examined more in future research.

### Multidisciplinary Interventions for DCD

There are several training programs frequently utilized for treating specific motor features of DCD used by occupational and physical therapists such as Cognitive Orientation to Daily Occupational Performance (CO-OP) and Neuromotor Task Training (Smits-Engelsman, 2013; Smits-Engelsman et al., 2018). CO-OP and NTT are activity or task-oriented approaches which specifically target physical fitness and motor task-performance (Montgomery et al., 2018) and are historically effective for treating children with DCD (Polatajko & Mandich, 2004). These trainings, along with any other existing treatment, are not intended to cure DCD, and can substantially help the patient improve specific motor skills. However, the increasing evidence that DCD is more than just a disorder of motor functions qualifies that more psychological interventions should be comprehensively investigated (Tamplain & Miller, 2021). It is possible that psychological support may be equally important as physical treatment for some patients with DCD. This is especially relevant to reduce any risk for potential secondary psychosocial consequences such as depression or anxiety (Kirby et al., 2013).

Presently, an interdisciplinary approach along with occupational therapists and physiotherapists (e.g., typical treatment: CO-OP; NTT for training specific motor skills) is recommended for effective intervention with DCD (Blank et al., 2019; Montgomery et al., 2018). It is also important that specific difficulties to the individual and the goals of the patient are considered in treatment, as this has led to reduced anxiety compared to preset large-group interventions in children (Caçola et al., 2016). For example, one individual might find it most pertinent to practice typing on a keyboard for work or school, while another might want to reduce their anxiety participating in group sports. The role of motor concerns may be direct or indirect in treatment, but regardless, the patient’s preferences should determine the approach and prioritization of goals in their treatment plan. A recent review and meta-analysis of motor-based interventions for DCD also suggests that effective interventions are personalized for the patient and their specific goals, contexts, active involvement, functionality and support from peers (Smits-Engelsman et al., 2018). In sum, tailor-made treatments have potential to improve both motor and psychological outcomes, and psychological interventions for secondary problems and psychological consequences of DCD should be examined in great detail future research.

## Discussion

Returning to the case of Tanja, it is now clear the patient should be assessed for DCD, with consideration of potential co-occurring ADHD. It is important in her case, to identify if her attentional difficulties are linked to motor activity, in which case she may just have DCD. In psychotherapy, screening for secondary anxiety and depression and working on stress-management would be important for immediate action. A psychotherapist should also consider referrals to a physical or occupational therapist to work on specific motor skills training relevant to her work and school activities (e.g., practicing typing). With a collaborative and patient-focused approach, there is hope for Tanja to feel substantially less burdened by her motor and attentional difficulties.

Taken together, the recent research on DCD highlights several key areas of consideration for clinical psychologists in Europe. First, DCD is a complex disorder with motor-based symptoms, several probable secondary symptoms and psychological consequences (e.g., executive functions; anxiety; depression). These secondary impairments of DCD should continue to be examined systematically in all age groups, and with the consideration of co-occurring disorders. More specifically, the prevalence of DCD should be examined more thoroughly across Europe in adults and children to identify a more accurate prevalence rate that may exceed the presumed international rate of 5% (e.g., Delgado-Lobete et al., 2019). This research may function in parallel with the necessary validation of DCD screening tools in additional languages. Future research should also aim to identify if prevalence differs across genders, as well as the consistency of other links such as left-handedness (Darvik et al., 2018) and links to motor integration.

Second, more attention should be devoted to the co-occurrences with DCD, especially between DCD and ADHD. While some research has identified important differences between the two disorders (e.g., Goulardins et al., 2015), there is still ambiguity in the extent to which symptoms overlap and how this might impact co-occurrence rates. It has been suggested that one way to increase detection of DCD could be to screen for it in all potential ADHD cases, considering their high co-occurrence rate (Lange, 2018). Moreover, screening for DCD when at least one other neurodevelopmental condition is clearly present, especially ADHD, should be consistently practiced. Future research should also identify unique symptomatic profiles of DCD and ADHD, and researchers examining DCD or ADHD should consistently screen for the other disorder.

Third, additional attention should be given to the emerging adult and adult populations with DCD in research and practice. While it is possible to diagnose DCD in adults, there are few tools that can be used for the diagnostic process. Furthermore, while there is evidence of psychosocial problems in adulthood (Kirby et al., 2013) there is no research to explore the effects of psychotherapy among adults. While it is thought that the same core motor symptoms generally cross into adulthood (e.g., Kirby et al., 2010; Kirby et al., 2011), along with potential secondary psychological concerns (e.g., depression, anxiety; Kirby et al., 2013), there is a paucity of evidence on the manifestation of these difficulties in new contexts (e.g., transitioning to news schools or jobs). Future research should continue to build the evidence for symptom profiles and screening tools for adults, and more specifically, psychological interventions should be examined for effectiveness in all age groups.

Finally, evidence-based treatments for the primary symptoms and secondary problems are crucial to foster the improvement in quality of life for DCD patients. There is increasing evidence that the psychosocial sequelae of DCD can be addressed with elements of psychotherapy adjunct to motor therapies. Thus, treatment should be collaboratively tailored toward the individual needs of each patient (e.g., Smits-Engelsman et al., 2018). It may also be worth considering if other therapies may be relevant to the treatment of DCD, such as a familial approach in treatment that is often used for ADHD (Weyers et al., 2019). Future research should include a broader examination of the family and social system in the impact and treatment of DCD.

### Conclusion

Overall, there are existing research gaps in the understanding of DCD, however, a recent increase in international attention to the condition is promising. We deem it relevant that more European psychological researchers and practitioners take note of this upsurge and integrate motor skill screenings into their work where possible. Such inclusion is pertinent for more accurate symptom profiles, prevalence estimates, improved differential diagnosis, and effective treatment of the symptoms of DCD across all age groups.

## References

[r1] American Psychiatric Association. (2013). *Diagnostic and statistical manual of mental disorders* (5th ed.). American Psychiatric Publishing.

[r98] American Psychiatric Association. (2022). *Diagnostic and statistical manual of mental disorders* (5th ed., text rev.). 10.1176/appi.books.9780890425787

[r2] Baharudin, N. S., Harun, D., & Kadar, M. (2020). An assessment of the movement and function of children with specific learning disabilities: A review of five standardised assessment tools. The Malaysian Journal of Medical Sciences: MJMS, 27(2), 21–36. 10.21315/mjms2020.27.2.3 32788838 PMC7409574

[r78] Bar-Ilan Traub, R., Waldwan-Levi, A., & Parush, S. (2005). Validity and reliability of the Developmental Coordination Disorder Questionnaire for school-aged children in Israel. Israeli Society of Occupational Therapy*,* 14(4), E181-E183. https://www.jstor.org/stable/23468933

[r3] Barnett, A. L. (2014). Is there a “movement thermometer” for Developmental Coordination Disorder? Current Developmental Disorders Reports, 1(2), 132–139. 10.1007/s40474-014-0011-9

[r4] Bernardi, M., Leonard, H. C., Hill, E. L., Botting, N., & Henry, L. A. (2018). Executive functions in children with Developmental Coordination Disorder: A 2‐year follow‐up study. Developmental Medicine and Child Neurology, 60(3), 306–313. 10.1111/dmcn.13640 29238952

[r5] Bishop, D. V. M. (2010). Which neurodevelopmental disorders get researched and why? PLoS One, 5(11), e15112. 10.1371/journal.pone.0015112 21152085 PMC2994844

[r6] Blank, R., Barnett, A. L., Cairney, J., Green, D., Kirby, A., Polatajko, H., Rosenblum, S., Smits‐Engelsman, B., Sugden, D., Wilson, P., & Vinçon, S. (2019). International clinical practice recommendations on the definition, diagnosis, assessment, intervention, and psychosocial aspects of Developmental Coordination Disorder. Developmental Medicine and Child Neurology, 61(3), 242–285. 10.1111/dmcn.14132 30671947 PMC6850610

[r7] Blank, R., Jenetzky, E., & Vinçon, S. (2014). *Bruininks-Oseretzky Test der motorischen Fähigkeiten* (2nd ed.) [Bruininks-Oseretzky Test of motor skills, second edition – German]. Pearson.

[r8] Blank, R., Smits‐Engelsman, B., Polatajko, H., & Wilson, P. (2012). European Academy for Childhood Disability (EACD): Recommendations on the definition, diagnosis and intervention of Developmental Coordination Disorder (long version). Developmental Medicine and Child Neurology, 54(1), 54–93. 10.1111/j.1469-8749.2011.04171.x 22171930

[r9] Bruininks, R. H., & Bruininks, B. D. (2005). *Bruininks-Oseretsky test of motor proficiency* (2nd ed.). NFER-Nelson.

[r10] Caçola, P., Miller, H. L., & Williamson, P. O. (2017). Behavioral comparisons in Autism Spectrum Disorder and Developmental Coordination Disorder: A systematic literature review. Research in Autism Spectrum Disorders, 38, 6–18. 10.1016/j.rasd.2017.03.004 29057009 PMC5646683

[r11] Caçola, P., Romero, M., Ibana, M., & Chuang, J. (2016). Effects of two distinct group motor skill interventions in psychological and motor skills of children with Developmental Coordination Disorder: A pilot study. Disability and Health Journal, 9(1), 172–178. 10.1016/j.dhjo.2015.07.007 26329699

[r12] Cairney, J., Veldhuizen, S., King-Dowling, S., Faught, B. E., & Hay, J. (2017). Tracking cardiorespiratory fitness and physical activity in children with and without motor coordination problems. Journal of Science and Medicine in Sport, 20(4), 380–385. 10.1016/j.jsams.2016.08.025 27760715

[r13] Camilleri, L. M., Buhagiar, N., Misfud, C., & Bonello, M. (2020). Validating the Developmental Coordination Disorder Questionnaire for use with children aged between five and fifteen in the Maltese context. Malta Journal of Health Sciences, 7(1), 31–38. 10.14614/DEVCOORDDIS/6/20

[r14] Cancer, A., Minoliti, R., Crepaldi, M., & Antonietti, A. (2020). Identifying Developmental Motor Difficulties: A review of tests to assess motor coordination in children. Journal of Functional Morphology and Kinesiology, 5(1), 16. 10.3390/jfmk5010016 33467232 PMC7739297

[r15] Cantell, M., Houwen, S., & Schoemaker, M. (2019). Age-related validity and reliability of the Dutch Little Developmental Coordination Disorder Questionnaire (LDCDQ-NL). Research in Developmental Disabilities, 84, 28–35. 10.1016/j.ridd.2018.02.010 29477487

[r16] Capistrano, R., Ferrari, E. P., Souza, L. P. D., Beltrame, T. S., & Cardoso, F. L. (2015). Concurrent validation of the MABC-2 motor tests and MABC-2 checklist according to the Developmental Coordination Disorder Questionnaire–BR. Motriz: Journal of Physical Education, 21(1), 100–106. 10.1590/S1980-65742015000100013

[r17] Caravale, B., Baldi, S., Capone, L., Presaghi, F., Balottin, U., & Zoppello, M. (2015). Psychometric properties of the Italian version of the Developmental Coordination Disorder Questionnaire (DCDQ–Italian). Research in Developmental Disabilities, 36, 543–550. 10.1016/j.ridd.2014.10.035 25462515

[r18] Carlsson, T., Molander, F., Taylor, M., Jonsson, U., & Bölte, S. (2021). Early environmental risk factors for neurodevelopmental disorders – A systematic review of twin and sibling studies. Development and Psychopathology, 33(4), 1448–1495. 10.1017/S0954579420000620 32703331 PMC8564717

[r19] Cleaton, M. A. M., & Kirby, A. (2018). Why do we find it so hard to calculate the burden of neurodevelopmental disorders? Journal of Childhood & Developmental Disorders*,* 4(3), 10. 10.4172/2472-1786.100073

[r20] Conzelmann, A., Mucha, R. F., Jacob, C. P., Weyers, P., Romanos, J., Gerdes, A. B. M., Bähne, C. G., Boreatti-Hümmer, A., Heine, M., Alpers, G. W., Warnke, A., Fallgatter, A. J., Lesch, K.-P., & Pauli, P. (2009). Abnormal affective responsiveness in Attention-Deficit/Hyperactivity Disorder: Subtype differences. Biological Psychiatry, 65(7), 578–585. 10.1016/j.biopsych.2008.10.038 19100967

[r21] Darvik, M., Lorås, H., & Pedersen, A. V. (2018). The prevalence of left-handedness is higher among individuals with Developmental Coordination Disorder than in the general population. Frontiers in Psychology, 9, 1948. 10.3389/fpsyg.2018.01948 30405473 PMC6200842

[r22] Delgado-Lobete, L., Montes-Montes, R., Méndez-Alonso, D., & Prieto-Saborit, J. A. (2021). Cross-cultural adaptation and preliminary reliability of the Adolescents and Adults Coordination Questionnaire into European Spanish. International Journal of Environmental Research and Public Health, 18, 6405. 10.3390/ijerph18126405 34199221 PMC8296233

[r23] Delgado-Lobete, L., Santos-del-Riego, S., Pértega-Díaz, S., & Montes-Montes, R. (2019). Prevalence of suspected Developmental Coordination Disorder and associated factors in Spanish classrooms. Research in Developmental Disabilities, 86, 31–40. 10.1016/j.ridd.2019.01.004 30654220

[r24] Draghi, T. T. G., Neto, J. L. C., Rohr, L. A., Jelsma, L. D., & Tudella, E. (2020). Symptoms of anxiety and depression in children with Developmental Coordination Disorder: A systematic review. Jornal de Pediatria, 96(1), 8–19. 10.1016/j.jped.2019.03.002 31029680 PMC9432331

[r25] Ellinoudis, T., Evaggelinou, C., Kourtessis, T., Konstantinidou, Z., Venetsanou, F., & Kambas, A. (2011). Reliability and validity of age band 1 of the Movement Assessment Battery for Children – Second edition. Research in Developmental Disabilities*,* 32(3), 1046–1051. 10.1016/j.ridd.2011.01.035 21333488

[r26] Goulardins, J. B., Rigoli, D., Licari, M., Piek, J. P., Hasue, R. H., Oosterlaan, J., & Oliveria, J. A. (2015). Attention Deficit Hyperactivity Disorder and Developmental Coordination Disorder: Two separate disorders or do they share a common etiology. Behavioural Brain Research, 292, 484–492. 10.1016/j.bbr.2015.07.009 26168770

[r27] Harrowell, I., Hollén, L., Lingam, R., & Emond, A. (2018). The impact of Developmental Coordination Disorder on educational achievement in secondary school. Research in Developmental Disabilities, 72, 13–22. 10.1016/j.ridd.2017.10.014 29080482 PMC5770330

[r28] Henderson, S., Sugden, D., & Barnett, A. (2007). *Movement Assessment Battery for Children–2.* Pearson Assessment

[r29] Holm, I., Tveter, A. T., Aulie, V. S., & Stuge, B. (2013). High intra- and inter-rater chance variation of the Movement Assessment Battery for Children 2, Age band 2. Research in Developmental Disabilities, 34(2), 795–800. 10.1016/j.ridd.2012.11.002 23220056

[r30] Iwar, K. (2015). *The Developmental Coordination Disorder Questionnaire 2007: Test-retest av den svenska översättningen* [Master’s thesis, Swedish School of Sport and Health Sciences]. GIH Publication Database. http://urn.kb.se/resolve?urn=urn:nbn:se:gih:diva-3953

[r31] Jaščenoka, J., & Petermann, F. (2018). Umschriebene motorische Entwicklungsstörungen (UEMF): Weisen betroffene Kinder spezifische Intelligenzprofile auf? [Developmental Coordination Disorders: Do children have specific intelligence profiles?]. Kindheit und Entwicklung, 27(1), 14–30. 10.1026/0942-5403/a000241

[r32] Joshi, D., Missiuna, C., Hanna, S., Hay, J., Faught, B. E., & Cairney, J. (2015). Relationship between BMI, waist circumference, physical activity and probable Developmental Coordination Disorder over time. Human Movement Science, 40, 237–247. 10.1016/j.humov.2014.12.011 25617993

[r33] Kaiser, M. L., Schoemaker, M. M., Albaret, J. M., & Geuze, R. H. (2015). What is the evidence of impaired motor skills and motor control among children with Attention Deficit Hyperactivity Disorder (ADHD)? Systematic review of the literature. Research in Developmental Disabilities, 36, 338–357. 10.1016/j.ridd.2014.09.023 25462494

[r34] Kennedy-Behr, A., Wilson, B. N., Rodger, S., & Mickan, S. (2013). Cross-cultural adaptation of the Developmental Coordination Disorder Questionnaire 2007 for German-speaking countries: DCDQ-G. Neuropediatrics, 44(5), 245–251. 10.1055/s-0033-1347936 23716299

[r35] Kirby, A., Edwards, L., & Sugden, D. (2011). Emerging adulthood and Developmental Co-ordination Disorder. Journal of Adult Development, 18(3), 107–113. 10.1007/s10804-011-9123-1 21334175

[r36] Kirby, A., Edwards, L., Sugden, D., & Rosenblum, S. (2010). The development and standardization of the Adult Developmental Co-ordination disorders/dyspraxia checklist (ADC). Research in Developmental Disabilities, 31(1), 131–139. 10.1016/j.ridd.2009.08.010 19819107

[r37] Kirby, A., Williams, N., Thomas, M., & Hill, E. L. (2013). Self-reported mood, general health, wellbeing and employment status in adults with suspected DCD. Research in Developmental Disabilities, 34(4), 1357–1364. 10.1016/j.ridd.2013.01.003 23417140

[r38] Lange, S. M. (2018). ADHD and comorbid Developmental Coordination Disorder: Implications and recommendations for school psychologists. Contemporary School Psychology, 22(1), 30–39. 10.1007/s40688-017-0122-5

[r39] Leonard, H. C., & Hill, E. L. (2015). Executive difficulties in Developmental Coordination Disorder: Methodological issues and future directions. Current Developmental Disorders Reports, 2, 141–149. 10.1007/s40474-015-0044-8

[r40] Lichtenstein, P., Carlström, E., Råsta, M., Gillberg, C., & Anckarsäter, H. (2010). The genetics of autism spectrum disorders and related neuropsychiatric disorders in childhood. The American Journal of Psychiatry, 167(11), 1357–1363. 10.1176/appi.ajp.2010.10020223 20686188

[r41] Mancini, V., Rigoli, D., Roberts, L., & Piek, J. (2019). Motor skills and internalizing problems throughout development: An integrative research review and update of the environmental stress hypothesis research. Research in Developmental Disabilities, 84, 96–111. 10.1016/j.ridd.2018.07.003 30054197

[r42] Marquet-Doléac, J., Soppelsa, R., & Albaret, J. M. (2016). MABC-2 Batterie d’évaluation du mouvement chez l’enfant–2e édition–adaptation française [Movement Assessment Battery for Children–2, French adaptation]. Éditions du Centre de Psychologie Appliquée.

[r43] McLeod, K. R., Langevin, L. M., Goodyear, B. G., & Dewey, D. (2014). Functional connectivity of neural motor networks is disrupted in children with Developmental Coordination Disorder and Attention-Deficit/Hyperactivity Disorder. NeuroImage: Clinical, 4, 566–575. 10.1016/j.nicl.2014.03.010 24818082 PMC3984446

[r44] Meachon, E. J., Beitz, C., Zemp, M., Wilmut, K., & Alpers, G. W. (2022). The Adult Developmental Coordination Disorders/Dyspraxia Checklist – German: Adapted factor structure for the differentiation of DCD and ADHD. Research in Developmental Disabilities, 126, 104254. 10.1016/j.ridd.2022.104254 35550942

[r45] Meachon, E. J., Meyer, M., Wilmut, K., Zemp, M., & Alpers, G. W. (2021). Evoked potentials differentiate Developmental Coordination Disorder from Attention-Deficit/Hyperactivity Disorder in a stop-signal task: A pilot study. Frontiers in Human Neuroscience, 15, 629479. 10.3389/fnhum.2021.629479 33776670 PMC7990764

[r46] Milidou, I., Lindhard, M. S., Søndergaard, C., Olsen, J., & Henriksen, T. B. (2015). Developmental Coordination Disorder in children with a history of infantile colic. The Journal of Pediatrics, 167(3), 725–730.e2. 10.1016/j.jpeds.2015.06.005 26164380

[r47] Montgomery, I., Glegg, S., Boniface, G., & Zwicker, J. G. (2018). *Management of Developmental Coordination Disorder.* Children's & Women's Health Centre of British Columbia. http://www.childdevelopment.ca/E4PGroup/E4P.aspx

[r48] Niño-Cruz, G. I., Carmago-Lemos, D. M., Velásquez-Escobar, L. I., Rodríguez-Ortiz, J. K., & Patiño-Segura, M. S. (2019). Batería para la evaluación del movimiento en niños–2– banda 1. Confiabilidad de la versión en español [Movement Assessment Battery for Children–2– band 1: Validity of the Spanish version]. Revista Chilena de Pediatria, 90(5), 522–532. 10.32641/rchped.v90i5.881 31859736

[r97] Moret, J., Pirson, J., & Van Der Massen, E. (2019). *Psychometric properties of the Flemish Little Developmental Coordination Disorder Questionnaire (L-DCD-Q-VL).* (Study, Ghent University). https://libstore.ugent.be/fulltxt/RUG01/002/783/371/RUG01-002783371_2019_0001_AC.pdf

[r49] Nowak, A. (2016). Cross-cultural adaptation of the Developmental Coordination Disorder Questionnaire (DCDQ’07) for the population of Polish children. Biomedical Human Kinetics, 8(1), 17–23. 10.1515/bhk-2016-0003

[r50] Okuda, P. M. M., Pangelinan, M., Capellini, S. A., & Cogo-Moreira, H. (2019). Motor skills assessments: Support for a general motor factor for the Movement Assessment Battery for Children–2 and the Bruininks-Oseretsky Test of motor proficiency–2. Trends in Psychiatry and Psychotherapy, 41(1), 51–59. 10.1590/2237-6089-2018-0014 30994783

[r51] Omer, S., Jijon, A. M., & Leonard, H. C. (2019). Research Review: Internalising symptoms in Developmental Coordination Disorder: A systematic review and meta‐analysis. Journal of Child Psychology and Psychiatry, and Allied Disciplines, 60(6), 606–621. 10.1111/jcpp.13001 30485419 PMC7379561

[r52] Paquet, A., Olliac, B., Golse, B., & Vaivre-Douret, L. (2019). Nature of motor impairments in Autism Spectrum Disorder: A comparison with Developmental Coordination Disorder. Journal of Clinical and Experimental Neuropsychology, 41(1), 1–14. 10.1080/13803395.2018.1483486 29923455

[r53] Pearsall-Jones, J. G., Piek, J. P., Rigoli, D., Martin, N. C., & Levy, F. (2009). An investigation into etiological pathways of DCD and ADHD using a monozygotic twin design. Twin Research and Human Genetics, 12(4), 381–391. 10.1375/twin.12.4.381 19653839

[r54] Petermann, F. (Ed.). (2008). *Movement Assessment Battery for Children–2 – Deutsche Fassung*, Pearson.

[r55] Polatajko, H. J., & Mandich, A. (2004). *Enabling occupation in children: The Cognitive Orientation to daily Occupational Performance (CO-OP) approach*. CAOT Publications ACE.

[r56] Prado, M. S. S., Magalhães, L. C., & Wilson, B. N. (2009). Cross-cultural adaptation of the Developmental Coordination Disorder Questionnaire for Brazilian children. Brazilian Journal of Physical Therapy, 13(3), 236–243. 10.1590/S1413-35552009005000024

[r57] Psotta, R., Hendl, J., Fromel, K., & Lehnert, M. (2012). The second version of the Movement Assessment Battery for Children: A comparative study in 7-10 year old children from the Czech Republic and the United Kingdom. Acta Gymnica, 42(4), 19–27. 10.5507/ag.2012.020

[r58] Purcell, C., Scott-Roberts, S., & Kirby, A. (2015). Implications of DSM-5 for recognising adults with Developmental Coordination Disorder (DCD). British Journal of Occupational Therapy, 78(5), 295–302. 10.1177/0308022614565113

[r59] Ray-Kaeser, S., Thommen, E., Martini, R., Jover, M., Gurtner, B., & Bertrand, A. M. (2019). Psychometric assessment of the French European Developmental Coordination Disorder Questionnaire (DCDQ-FE). PLoS One, 14(5), e0217280. 10.1371/journal.pone.0217280 31120966 PMC6532915

[r60] Rigoli, D., & Piek, J. P. (2016). Motor problems as a risk factor for poorer mental health in children and adolescents: What do we know and should we be screening for psychological difficulties in those with poor motor skills? Current Developmental Disorders Reports, 3, 190–194. 10.1007/s40474-016-0091-9

[r61] Rihtman, T., Wilson, B. N., Cermak, S., Rodger, S., Kennedy-Behr, A., Snowdon, L., Schoemaker, M., Cantell, M., Houwen, S., Jover, M., Albaret, J., Ray-Kaeser, L., Magalhāes, L., Cardoso, L., Waelvelde, H. V., Hultsch, D., Vinçon, S., Tseng, M., Pienaar, A. E, … Parush, S. (2015). Can a little instrument make a big noise? A cross-cultural collaboration for identifying motor delay in young preschoolers. Journal of Multimorbidity and Comorbidity, 5(2), 32–109. 10.15256/joc.2015.5.52

[r62] Rihtman, T., Wilson, B. N., & Parush, S. (2011). Development of the Little Developmental Coordination Disorder Questionnaire for preschoolers and preliminary evidence of its psychometric properties in Israel. Research in Developmental Disabilities, 32(4), 1378–1387. 10.1016/j.ridd.2010.12.040 21295440

[r63] Salamanca, L. M., Naranjo, M. M. C., & González, A. P. (2012). Traducción al español del cuestionario para diagnostic de trastorno del desarrolla de la coordinaciòn [Spanish translation of the questionnaire to diagnose Developmental Coordination Disorder]. Revistas Ciencias de la Salud*,* 10(2), 195-206. https://www.redalyc.org/home.oa

[r64] Sartori, R. F., Valentini, N. C., & Fonseca, R. P. (2020). Executive function in children with and without Developmental Coordination Disorder: A comparative study. Child: Care, Health and Development, 46(3), 294–302. 10.1111/cch.12734 31845379

[r65] Schoemaker, M. M., Flapper, B., Verheij, N. P., Wilson, B. N., Reinders-Messelink, H. A., & de Kloet, A. (2006). Evaluation of the Developmental Coordination Disorder Questionnaire (DCDQ) as a screening instrument. Developmental Medicine and Child Neurology, 48(8), 668–673. 10.1017/S001216220600140X 16836779

[r66] Schoemaker, M. M., Niemeijer, A. S., Flapper, B. C. T., & Smits-Engelsman, B. C. M. (2012). Validity and reliability of the Movement Assessment Battery for Children–2 checklist for children with and without motor impairments. Developmental Medicine and Child Neurology, 54(4), 368–375. 10.1111/j.1469-8749.2012.04226.x 22320829

[r67] Serrano-Gómez, M. E., & Correa-Bautista, J. E. (2015). Propiedades psicométricas del test de competencias motoras bruininks oseretsky en versión corta para niños entre 4 y 7 años en Chía y Bogotá, D. C., Colombia [Psychometric properties of the short form of the Bruininks-Oseretsky Test of motor proficiency in children between 4 and 7 years in Chía and Bogotá – Colombia]. Revista de la Facultad de Medicina, 63(4), 633–640. 10.15446/revfacmed.v63.n4.49965

[r68] Smits-Engelsman, B., Vinçon, S., Blank, R., Quadrado, V. H., Polatajko, H., & Wilson, P. (2018). Evaluating the evidence for motor-based interventions in Developmental Coordination Disorder: A systematic review and meta-analysis. Research in Developmental Disabilities, 74, 72–102. 10.1016/j.ridd.2018.01.002 29413431

[r69] Smits-Engelsman, B. (2013). Neuromotor task training – zum motorischen Lernen befähigen [Neuromotor task training – Enabling motor learning]. ergopraxis, 6(9), 24–30. 10.1055/s-0033-1356910

[r70] Spittle, A. J., Dewey, D., Nguyen, T.-N.-N., Ellis, R., Burnett, A., Kwong, A., Lee, K., Cheong, J. L. Y., Doyle, L. W., & Anderson, P. J. (2021). Rates of Developmental Coordination Disorder in children born very preterm. The Journal of Pediatrics, 231, 61–67.e2. 10.1016/j.jpeds.2020.12.022 33340547

[r71] Tal Saban, M., & Kirby, A. (2018). Adulthood in Developmental Coordination Disorder (DCD): A review of current literature based on ICF perspective. Motor Disorders, 5(1), 9–17. 10.1007/s40474-018-0126-5

[r72] Tal Saban, M., Ornoy, A., Grotto, I., & Parush, S. (2012). Adolescents and Adults Coordination Questionnaire: Development and psychometric properties. The American Journal of Occupational Therapy, 66(4), 406–413. 10.5014/ajot.2012.003251 22742688

[r73] Tamplain, P., & Miller, H. L. (2021). What can we do to promote mental health among individuals with Developmental Coordination Disorder? Current Developmental Disorders Reports, 8, 24–31. 10.1007/s40474-020-00209-7 34306965 PMC8297602

[r74] Targum, S. D., & Adler, L. A. (2014). Our current understanding of adult ADHD. Innovations in Clinical Neuroscience, 11(11-12), 30–35. 25621186 PMC4301030

[r75] Tercon, J., Rihtman, T., & Wilson, B. N. (2015). Abstracts: 11th international conference on Developmental Coordination Disorder (DCD-11): Developmental coordination disorder and other neurodevelopmental disorders: A focus on comorbidity. Journal of Comorbidity, 5(2), 32–109. 10.15256/joc.2015.5.52

[r76] Thomas, M., & Christopher, G. (2018). Fatigue in Developmental Coordination Disorder: An exploratory study in adults. Fatigue: Biomedicine, Health & Behavior, 6(1), 41–51. 10.1080/21641846.2018.1419564

[r77] Timler, A., McIntyre, F., & Hands, B. (2018). Adolescents’ self-reported motor assessments may be more realistic than those of their parents. British Journal of Occupational Therapy, 81(4), 227–233. 10.1177/0308022617743681

[r79] Valentini, N. C., Olivera, M. A., Pangelinan, M. M., Whitall, J., & Clark, J. E. (2017). Can the MABC discriminate and predict motor impairment? A comparison of Brazilian and North American children. International Journal of Therapy and Rehabilitation, 24(3), 105–113. 10.12968/ijtr.2017.24.3.105

[r80] Valentini, N. C., Ramalho, M. H., & Oliveira, M. A. (2014). Movement Assessment Battery for Children–2: Translation, reliability, and validity for Brazilian children. Research in Developmental Disabilities, 35(3), 733–740. 10.1016/j.ridd.2013.10.028 24290814

[r81] van Hoorn, J. F., Schoemaker, M. M., Stuive, I., Dijkstra, P. U., Rodrigues Trigo Pereira, F., van der Sluis, C. K., & Hadders-Algra, M. (2021). Risk factors in early life for Developmental Coordination Disorder: A scoping review. Developmental Medicine and Child Neurology, 63(5), 511–519. 10.1111/dmcn.14781 33345317 PMC8048603

[r82] Vinçon, S., Green, D., Blank, R., & Jenetzky, E. (2017). Ecological validity of the German Bruininks-Oseretsky Test of motor proficiency – 2nd edition. Human Movement Science*,* 53, 45-54. 10.1016/j.humov.2016.10.005 27832925

[r83] Weyers, L., Zemp, M., & Alpers, G. W. (2019). Impaired interparental relationships in families of children with Attention-Deficit/Hyperactivity Disorder (ADHD). Zeitschrift für Psychologie mit Zeitschrift für Angewandte Psychologie, 227(1), 31–41. 10.1027/2151-2604/a000354

[r84] Wilmut, K. (2017). Performance under varying constraints in Developmental Coordination Disorder (DCD): Difficulties and compensations. Current Developmental Disorders Reports, 4(2), 46–52. 10.1007/s40474-017-0108-z

[r85] Wilmut, K., & Purcell, C. (2020). The lived experience of crossing the road when you have Developmental Coordination Disorder (DCD): The perspectives of parents of children with DCD and adults with DCD. Frontiers in Psychology, 11, 587042. 10.3389/fpsyg.2020.587042 33329244 PMC7710519

[r86] Wilson, B. N., Crawford, S. G., Green, D., Roberts, G., Aylott, A., & Kaplan, B. J. (2009). Psychometric properties of the revised Developmental Coordination Disorder Questionnaire. Physical & Occupational Therapy in Pediatrics, 29(2), 182–202. 10.1080/01942630902784761 19401931

[r87] Wilson, B. N., Creighton, D., Crawford, S. G., Heath, J. A., Semple, L., Tan, B., & Hansen, S. (2015). Psychometric properties of the Canadian Little Developmental Coordination Disorder Questionnaire for preschool children. Physical & Occupational Therapy in Pediatrics, 35(2), 116–131. 10.3109/01942638.2014.980928 25456610

[r88] Wilson, B. N., Neil, K., Kamps, P. H., & Babcock, S. (2013). Awareness and knowledge of Developmental Co-ordination Disorder among physicians, teachers, and parents. Child: Care, Health and Development, 39(2), 296–300. 10.1111/j.1365-2214.2012.01403.x 22823542 PMC3579234

[r89] Wilson, P. H., Ruddock, S., Rahimi-Golkhandan, S., Piek, J., Sugden, D., Green, D., & Steenbergen, B. (2020). Cognitive and motor function in Developmental Coordination Disorder. Developmental Medicine and Child Neurology, 62(11), 1317–1323. 10.1111/dmcn.14646 32770756

[r90] World Health Organization. (2016). *International statistical classification of diseases and related health problems* (10th ed.). https://icd.who.int/browse10/2016/en 10.2471/BLT.25.294650 PMC13531092 42683092

[r91] World Health Organization. (2020). *International statistical classification of diseases and related health problems* (11th ed.). https://icd.who.int/10.2471/BLT.25.294650 PMC13531092 42683092

[r92] Yildirim, C. K., Altunalan, T., Acar, G., Elbasan, B., & Gucuyener, K. (2019). Cross-cultural adaptation of the Developmental Coordination Disorder Questionnaire in Turkish children. Perceptual and Motor Skills, 126(1), 40–49. 10.1177/0031512518809161 30428280

[r93] Young, S., Adamo, N., Ásgeirsdóttir, B. B., Branney, P., Beckett, M., Colley, W., Cubbin, S., Deeley, Q., Farrag, E., Gudjonsson, G., Hill, P., Hollingdale, J., Kilic, O., Lloyd, T., Mason, P., Paliokosta, E., Perecherla, S., Sedgwick, J., Skirrow, C., . . . Woodhouse, E. (2020). Females with ADHD: An expert consensus statement taking a lifespan approach providing guidance for the identification and treatment of Attention-Deficit/ Hyperactivity Disorder in girls and women. BMC Psychiatry, 20(1), 404. 10.1186/s12888-020-02707-9 32787804 PMC7422602

[r94] Zoia, S., Biancotto, M., Guicciardi, M., Lecis, R., Lucidi, F., Pelamatti, G. M., Carrozzi, M., Skabar, A., Sugden, D. A., Barnett, A. L., & Henderson, S. E. (2019). An evaluation of the Movement ABC-2 test for use in Italy: A comparison of data from Italy and the UK. Research in Developmental Disabilities, 84, 43–56. 10.1016/j.ridd.2018.04.013 29716782

[r95] Zwicker, J. G., Missiuna, C., Harris, S. R., & Boyd, L. A. (2012). Developmental Coordination Disorder: A review and update. European Journal of Paediatric Neurology, 16(6), 573–581. 10.1016/j.ejpn.2012.05.005 22705270

[r96] Zwicker, J. G., Suto, M., Harris, S. R., Vlasakova, N., & Missuna, C. (2018). Developmental Coordination Disorder is more than a motor problem: Children describe the impact of daily struggles on their quality of life. British Journal of Occupational Therapy, 81(2), 65–73. 10.1177/0308022617735046

